# Irradiation-induced hair graying in mice: an experimental model to evaluate the effectiveness of interventions targeting oxidative stress, DNA damage prevention, and cellular senescence

**DOI:** 10.1007/s11357-023-01042-7

**Published:** 2024-01-06

**Authors:** Anna Ungvari, Tamas Kiss, Rafal Gulej, Stefano Tarantini, Boglarka Csik, Andriy Yabluchanskiy, Peter Mukli, Anna Csiszar, Melissa L. Harris, Zoltan Ungvari

**Affiliations:** 1 Department of Public Health, Semmelweis University, Budapest, Hungary; 2 International Training Program in Geroscience, Doctoral School of Basic and Translational Medicine/Department of Public Health, Semmelweis University, Budapest, Hungary; 3 Vascular Cognitive Impairment, Neurodegeneration and Healthy Brain Aging Program, Department of Neurosurgery, University of Oklahoma Health Sciences Center, Oklahoma City, OK USA; 4 Oklahoma Center for Geroscience and Healthy Brain Aging, University of Oklahoma Health Sciences Center, Oklahoma City, OK USA; 5 First Department of Pediatrics, Semmelweis University, Budapest, Hungary; 6 Eötvös Loránd Research Network and Semmelweis University (ELKH-SE) Cerebrovascular and Neurocognitive Disorders Research Group, Budapest, Hungary; 7 Stephenson Cancer Center, University of Oklahoma, Oklahoma City, OK USA; 8 Department of Health Promotion Sciences, College of Public Health, University of Oklahoma Health Sciences Center, Oklahoma City, OK USA; 9 International Training Program in Geroscience, Doctoral School of Basic and Translational Medicine/Department of Translational Medicine, Semmelweis University, Budapest, Hungary; 10 Department of Biology, University of Alabama at Birmingham, Birmingham, AL USA

**Keywords:** Hair graying, Aging, Skin, Senescence, Canities, Achromotrichia

## Abstract

Hair graying, also known as canities or achromotrichia, is a natural phenomenon associated with aging and is influenced by external factors such as stress, environmental toxicants, and radiation exposure. Understanding the mechanisms underlying hair graying is an ideal approach for developing interventions to prevent or reverse age-related changes in regenerative tissues. Hair graying induced by ionizing radiation (γ-rays or X-rays) has emerged as a valuable experimental model to investigate the molecular pathways involved in this process. In this review, we examine the existing evidence on radiation-induced hair graying, with a particular focus on the potential role of radiation-induced cellular senescence. We explore the current understanding of hair graying in aging, delve into the underlying mechanisms, and highlight the unique advantages of using ionizing-irradiation–induced hair graying as a research model. By elucidating the molecular pathways involved, we aim to deepen our understanding of hair graying and potentially identify novel therapeutic targets to address this age-related phenotypic change.

## Introduction

Hair graying, also known as canities or achromotrichia, is a common age-related phenomenon characterized by the loss of pigmentation in hair strands [1, 2]. It is a result of a decrease in melanin production and distribution within the hair follicles [1–4]. Hair graying is a widespread occurrence, affecting both men and women as they grow older. Epidemiological studies have shown that the prevalence of hair graying increases with age, with a substantial proportion of individuals experiencing some degree of graying by their 50s or 60s. While hair graying is predominantly associated with chronological aging, there is considerable variation, both inter-individual and based on genetic background, in the onset and progression of graying [5, 6]. Hair graying is a multifaceted process influenced by a combination of genetic, environmental, and age-related factors [7–12].

In addition to the physiological changes associated with hair pigmentation that can inform on the molecular and cellular basis for aging, hair graying can have significant psychological and emotional impacts. For many individuals, the graying of hair is not merely a cosmetic concern but is intertwined with their self-perception and body image. Hair graying can lead to feelings of embarrassment, self-consciousness, and a perceived loss of attractiveness. These emotional problems associated with hair graying can have a detrimental effect on individuals’ self-esteem and overall well-being. Particularly for women, gray hair is one of the key physical traits that fuels age-related discrimination [13–16]. Therefore, understanding the mechanisms underlying hair graying and exploring potential interventions can not only address questions on the biological aspects of aging but also provide means for improving the emotional and psychological health of affected individuals.

In addition to the natural process of aging, several external factors have been identified as potential contributors to hair graying [8, 10, 17, 18]. These factors include environmental exposures, stress, and certain medical treatments. Among these factors, radiation exposure (IR) has gained significant attention due to its potential to induce DNA damage, oxidative stress, and cellular senescence in melanocytes. Understanding the mechanisms through which radiation exposure influences hair graying can provide valuable insights into developing strategies to prevent or reverse the effects of DNA damage associated with both radiation exposure and aging on hair pigmentation.

In recent years, there has been a growing body of preclinical evidence suggesting that the plasticity of aging can be harnessed, as demonstrated by the ability of various anti-aging treatments to delay or even reverse age-related changes in various organs, including the skin and fur of rodent models [19–21]. Various medications that cause hair repigmentation to occur in humans suggest that this plasticity is not limited to rodents [22, 23]. In addition, animal models of induced hair graying have emerged as particularly useful tools for evaluating the effectiveness of diverse anti-aging treatments aimed at reversing hair graying and promoting hair rejuvenation [24]. These models allow researchers to explore the potential of different interventions and therapies in a controlled setting, providing valuable insights into the feasibility and efficacy of anti-aging strategies for restoring hair pigmentation.

In this comprehensive review, we explore the current evidence surrounding ionizing-irradiation–induced hair graying. By synthesizing existing research, we aim to shed light on the mechanisms underlying this phenomenon, thereby contributing to our broader understanding of hair graying due to aging and exposure to external stressors, including radiation. Additionally, we highlight the advantages of using ionizing-irradiation–induced hair graying as an experimental model, specifically to address the role of cellular senescence and senolytics in hair graying, providing a roadmap for future investigations of potential therapeutic interventions.

## Hair graying: mechanisms and pathways

The process of hair pigmentation is complex and involves a delicate balance of various cellular and molecular mechanisms [25, 26]. Here, we provide a brief overview of cellular mechanisms that have been causally linked to hair graying [1, 2, 10, 27, 28].

### Melanogenesis and pigmentation

Melanogenesis plays a crucial role in hair pigmentation [25, 26]. The activity of melanocytes within the hair follicles and the transfer of melanin to hair shafts are essential for maintaining normal hair color. Melanocytes are specialized pigment-producing cells located in the hair follicles, which are responsible for the synthesis and distribution of melanin. Melanin exists in two main forms: eumelanin, which produces brown to black colors, and pheomelanin, which produces yellow to red colors. The balance between these two types of melanin, as well as the amount of melanin produced, determines the specific hair color observed. The process of melanogenesis is regulated by a complex interplay of genetic, epigenetic, cellular, and molecular mechanisms [25, 26, 29]. Key proteins involved in melanin synthesis include tyrosinase, tyrosinase-related protein 1 (TYRP1), and tyrosinase-related protein 2 (TRP2) [25, 26]. These enzymes catalyze the conversion of the amino acid tyrosine into melanin precursors, such as dopaquinone and dopachrome, which eventually lead to the production of mature melanin pigments. The biochemical reaction of melanin synthesis occurs within the melanosome, an intracellular organelle specialized for pigment production, and requires other proteins to promote and regulate melanosome maturation. This includes, but is not limited to, the formation of premelanosome-based proteinaceous fibrils as the scaffold for melanin deposition and the shuttling of ion channels to the melanosome membrane for intralumenal deacidification required for melanocyte enzyme function [30, 31]. The activity of melanocytes is regulated by various signaling pathways and factors. One crucial regulator is the microenvironment within the hair follicle, including factors secreted by adjacent cells, such as keratinocytes and dermal papilla cells [32–36]. Signal molecules, such as melanocyte-stimulating hormone (MSH), stem cell factor (SCF), endothelin-1, and WNT, are involved in modulating melanocyte function and melanogenesis during hair growth [37–43]. Mature melanosomes are transferred from melanocytes to the cortical and medullary keratinocytes of the hair shaft, imparting color to the hair. Melanosome transfer and distribution to skin and hair keratinocytes require various transport proteins and structural components, but the precise mechanisms are still debated.

Disruptions in melanogenesis and pigmentation processes can lead to hair graying. With aging, the number and function of melanocytes decrease, resulting in reduced melanin production and distribution [1, 2, 27, 32, 44]. Preclinical studies, primarily in rodents, suggest that an important cause for hair graying is the defective self-maintenance of melanocyte stem cells [27]. The resulting decline in melanin content leads to a loss of hair color and the appearance of gray or white hair. Genetic factors, environmental exposures, oxidative stress, and inflammation can also influence melanocyte function and melanin synthesis, contributing to premature hair graying.

### Human versus mouse hair graying

Both human and mouse hair undergoes cyclical regeneration. Anagen represents the growth phase of the hair follicle and is when the hair is formed. During anagen, melanocyte stem cells divide to give rise to melanocyte precursors that colonize the bulb of the hair as it extends proximally. Upon differentiation, these melanocytes deposit pigmented melanosomes into the matrix keratinocytes that create the elongating hair shaft. These differentiated matrix melanocytes comprise the hair follicle pigmentary unit (HFPU). Within anagen hairs in mouse, melanocyte stem cells reside within the hair bulge, a region anatomically distinct from the hair bulb [45]. Melanocytes and melanocyte stem cells are positioned similarly in human anagen hair follicles, but these follicles also harbor a population of amelanotic melanocytes, presumed melanocyte stem cells, in the outer root sheath and periphery of the hair bulb [3, 46, 47]. After the cessation of anagen, the lower portion of the hair follicle, including the hair bulb keratinocytes and its differentiated melanocytes, regresses in a programmed apoptotic process known as catagen [48, 49]. From catagen, hairs transition into telogen, a non-proliferative and quiescent stage [50, 51]. At telogen, only undifferentiated melanocyte stem cells are retained within the hair follicle.

Major differences exist between mouse and human hair cycling, particularly when considering human scalp hairs. The length of the growth phase for scalp hair is generally between 0.5 and 7 years but ranges significantly between individuals and between hairs on the head of the same individual [52]. The resting phase (which presumably includes catagen and telogen) can last 4–10 months. Using averages from an individual study of male subjects, this equates to human scalp hairs spending 68% of their time in anagen [52]. In mouse, this is reversed with hairs progressing through anagen quickly, ~ 13 days or ~ 16% of the average mouse hair cycle [53]. These differences are particularly meaningful when considering the etiology of hair graying in mouse versus human. Since the discovery of melanocyte stem cells in mouse [27], hair graying has been largely attributed to a stem cell defect. With age and in acute mouse models of hair graying, depletion of melanocyte stem cells is a common feature and in many cases is preceded by their premature and ectopic differentiation into melanocytes in the hair bulge [27, 54]. However, the short hair growth phase in mouse means that hair bulb melanocytes in this species are only required transiently to pigment the hair shaft. This leaves almost no opportunity for the HFPU to manifest time-dependent changes in their function in this model system. Conversely, human hairs that experience continuous growth over years can acquire bulb melanocytes with dysmorphic features, reduced dendricity, and positive staining for apoptosis markers [55]. This indicates local depletion of bulb melanocytes as a mechanism for graying in humans. While both melanocyte stem cells and hair bulb melanocytes decline in number with age in humans, it has been suggested that defects in stem cell–based regeneration of the HFPU are secondary to the decline of bulb melanocytes, and it is the latter that drives the progressive hair graying phenotype in humans.

### Mechanisms for hair graying: genetic and epigenetic factors

Genetic factors play a significant role in determining hair pigmentation, including the process of hair graying [56]. Several genes involved in melanin synthesis, regulation, and distribution have been identified as key contributors to hair graying [54, 56–63]. Genes associated with melanogenesis, such as the melanocortin 1 receptor (*MC1R*), tyrosinase-related protein 1 (*TYRP1*), and tyrosinase (*TYR*), have been found to influence hair pigmentation [39, 44, 64–66]. Variations or mutations in these genes can impact melanin production and distribution within the hair follicles, potentially leading to hair graying. Other genes involved in melanosome transport, such as the Myosin Va (*MYO5A*) gene, have also been implicated in hair graying [67–69]. Furthermore, genes related to melanocyte development and regulation, such as the paired box gene 3 (*PAX3*) [70], endothelin receptor type B (*EDNRB*) [43], and forkhead box protein D3 (*FOXD3*), SRY-box transcription factor 10 (*SOX10*), and melanocyte inducing transcription factor (*MITF*) [27, 54, 63], have been associated with hair graying. These genes contribute to the development and maintenance of hair follicles and their interaction with melanocytes, influencing melanin production and hair pigmentation.

In humans, the only gene with an established association with progressive, age-related hair graying is interferon regulatory factor 4 (*IRF4).* The intronic SNP rs12203592 serves a melanocyte-specific enhancer element that controls the expression of *IRF4*, and IRF4 protein cooperates with MITF to regulate the expression of the key melanogenic enzyme gene *Tyr.* The rs12203592 SNP is also associated with lighter hair color in humans and conditional knockout of *Irf4* in the melanocyte lineage in mice results in notably lighter coat color [71, 72]. Despite its role in pigmentation, it is currently unclear how IRF4 contributes to the age-related graying phenotype.

In addition to genetic factors, epigenetic modifications have also been implicated in the regulation of hair graying. Epigenetic modifications, including DNA methylation, histone modifications, and non-coding RNA molecules, are known to regulate critical cellular processes of aging [73] and likely also contribute to hair graying. Epigenetic changes can affect the activity of genes involved in melanogenesis and melanocyte function, among others [29]. Epigenetic modifications can be influenced by various factors that are known to modulate aging processes, including environmental exposures, lifestyle choices, and exposure to a wide range of stressors [29]. Further research is needed to uncover additional genetic and epigenetic factors associated with hair graying and to elucidate the specific mechanisms through which they influence melanocyte function and melanin production. By expanding our knowledge of the genetic and epigenetic basis of hair graying, we can gain a deeper understanding of this process and potentially identify targets for interventions aimed at preventing or reversing hair graying.

### Mechanisms for hair graying: neuronal regulation

The role of neuronal mechanisms in hair graying has garnered attention in recent research exploring the complex interplay between the nervous system and hair follicles [12, 74]. Empirical and anecdotal evidence has associated increased level of stress with premature hair graying [75]. Recent experimental evidence provide proof-of-concept showing that, in mouse models, acute stress leads to hair graying via a mechanism that involves exhaustion of melanocyte stem cells [12, 74]. Dermatomes, which are specific regions of skin innervated by a single spinal nerve, have been implicated in focal dysregulation of hair pigmentation (e.g., in segmental vitiligo [76, 77]). Neuronal signaling from the central nervous system to the hair follicles can influence melanocyte function and melanin production [12]. Studies suggest that neuronal factors, including neurotransmitters and neuropeptides, play a role in modulating melanocyte activity and regulating hair pigmentation [78–80]. Disruptions in the neural pathways and impaired communication between the nervous system and hair follicles may contribute to the loss of melanocyte function and premature hair graying. Importantly, there is proteomic evidence that hair graying associated with psychological stressors and its underlying mechanisms are potentially reversible [75]. Further research is needed to elucidate the precise mechanisms underlying the role of dermatomes and neuronal signaling in hair graying and how much these mechanisms ultimately contribute to human hair graying.

### Mechanisms for hair graying: oxidative stress, DNA damage, and mitochondrial dysfunction

Age-related oxidative stress, DNA damage, and mitochondrial dysfunction have emerged as significant factors in the process of hair graying [55, 81]. Oxidative stress directly affects melanocytes within the hair follicles [55, 81–83]. Reactive oxygen species (ROS) can cause damage to cellular components, including DNA, proteins, and lipids, and induce gene expression changes leading to cellular dysfunction and decreased melanin production [82, 83]. Accumulating evidence suggests that increased ROS production and impaired antioxidant defense systems contribute to melanocyte dysfunction and reduced melanin production. In comparison to pigmented hairs, melanocytes in human gray hairs exhibit high levels of 8OHdG, a free radical-induced DNA lesion that serves as a biomarker of oxidative stress. Gray hairs also exhibit a higher prevalence of the mitochondrial common deletion, an indication of mitochondrial oxidative damage, along with reduced catalase, methionine sulfoxide reductase and hydroxy-radical scavenging activity, and accumulation of hydrogen peroxide (H_2_0_2_) and oxidized amino acids. One direct target of this prooxidant environment is likely the melanogenic enzyme tyrosinase; H_2_0_2_-mediated oxidation of tyrosinase abolishes its activity [55, 84, 85]. Sources of reactive oxygen species can be endogenous or exogenous. Superoxide anion and H_2_0_2_ are both generated during melanin synthesis, and, despite this process being sequestered to the melanosome, these ROS are thought to contribute to melanocytes succumbing to oxidative challenge. UV and ionizing irradiation, toxins (like smoking), chemicals, and the inflammatory process are all external sources of ROS [86–88]. Melanin itself scavenges ROS and while cellular pigmentation is protective against UV and H_2_0_2_-induced damage, a melanocyte’s ability to balance its anti- and pro-oxidant properties is essential for preventing pathogenesis.

Damage to nuclear and mitochondrial DNA is a direct result of oxidative stress, and faulty mechanisms to mitigate this damage can lead to hair graying. ATM, a protein coded from the *ataxia-telangiectasia mutated gene* that senses and transduces DNA damage, is expressed specifically by human bulb melanocytes and positively correlates to pigmentation status; in hairs from middle aged individuals, lightly pigmented bulb melanocytes have reduced ATM in comparison to heavily pigmented bulb melanocytes. Human patients with ataxia-telangiectasia, a progeroid or accelerated-aging syndrome, experience several cutaneous progerias including hair graying. Similarly, *ATM*-deficient mice are significantly more sensitive to oxidative challenge. ATM knockout mice acquire a robust gray hair phenotype in response to low-level ionizing irradiation (3 Gy) because of melanocyte stem cell differentiation and depletion from the stem cell niche [83, 89, 90]. Gene expression studies on pigmented and non-pigmented hair follicles reveal downregulation of additional DNA repair mechanisms, namely a whole suite of nucleotide excision repair (NER) family genes, including *ERCC3*. Specific knockdown in melanocytes of *ERCC3*, a core DNA helicase subunit essential for unwinding DNA after damage for NER, reduces tyrosinase expression and activity [91]. Patients with Werner’s syndrome, 90% of which are attributed to gene mutations in the RecQ helicase gene *WRN* that functions in double strand break repair, also exhibit gray hair along with generalized progeria [92].

Age-related mitochondrial dysfunction can contribute to oxidative stress and further exacerbate melanocyte dysfunction and hair graying [2, 93–95]. Mitochondrial oxidative stress promotes the genesis of mtDNA damage in aging, and aging is associated with a decrease in mtDNA content and mitochondrial number. Modifications of the *POLG* gene, the alpha subunit of the mitochondrial DNA polymerase gamma, lead to a range of aging phenotypes, including hair graying. In mice, gray hair is observed in both PolG mtDNA-mutator mice that produce elevated mtDNA mutations due to disruption in the protein’s proofreading-exonuclease activity and PolG mtDNA-depleter mice that incur mitochondrial DNA depletion through a dominant negative mechanism [93, 96–98]. Interestingly, repletion of mtDNA in mtDNA depleter mice reverses many of their cutaneous aging phenotypes. This indicates that mtDNA depletion phenotypes are not necessarily permanent and thus amenable to therapeutics that restore mtDNA content levels. Interestingly, temporary transitions between pigmented and non-pigmented states occur naturally along individual human hairs, and proteomic analysis of white and black human hair follicles shows the upregulation of key genes involved in mitochondrial energy metabolism (*CPT1A, ACOT7, SOD1, CFL1, PGK1*) [75]. Altogether, these studies suggest active metabolic remodeling involving mitochondria is critical in the graying process, and there may be a key threshold where reversibility of these changes remains possible.

Understanding the role of oxidative stress, DNA damage, and mitochondrial dysfunction in hair graying provides insights into potential therapeutic targets aimed at reducing ROS levels, enhancing antioxidant defenses, and preserving melanocyte function. Mitigating oxidative stress through activation of the skin’s own antioxidant defense system via master regulator NRF2 provides one such example of promising strategies for preventing or reversing hair graying [99, 100].

## Radiation-induced hair graying to model aspects of age-related hair graying

Radiation exposure, particularly to ionizing radiation, causes premature hair graying and shows similar cellular outcomes as in age-related hair graying [90, 101–111]. Ionizing radiation increases the generation of reactive oxygen species (ROS) and DNA damage. Exposure to ionizing radiation can occur through various sources, including medical diagnostic procedures, radiation therapy for cancer treatment, occupational exposure, and accidental nuclear events.

### Historical perspective on radiation-induced effects

The association between radiation exposure and its effects on hair pigmentation has been recognized for almost a century. In the 1920s, interested in the biological action of X-rays, Hance and Murphy followed up an incidental observation of hair graying in rabbits during a study of X-ray dosage. Using mice, they observed that hairs with direct exposure to X-ray exhibit a pattern of delayed regrowth and emerge white. The amount of whitening correlated with length of X-ray exposure, and patches of hair that were completely white immediately after exposure remained white after a second round of hair regrowth [112]. In humans, significant observations were made in individuals exposed to ionizing radiation during medical treatments and nuclear accidents. One notable historical event was the atomic bombings of Hiroshima and Nagasaki in 1945 [103]. Survivors of the bombings, known as hibakusha, experienced various health effects, including changes in hair pigmentation. Many hibakusha reported a rapid onset of hair graying following exposure to high doses of radiation [103]. These observations provided early evidence of the connection between radiation exposure and hair graying.

### Clinical observations in radiation therapy patients

In addition to the atomic bombings, the field of radiation therapy also played a crucial role in understanding the effects of radiation on hair pigmentation. Radiation therapy is a widely used treatment modality for various types of cancer. While it is highly effective in targeting and eliminating cancer cells, it can also have unintended effects on healthy tissues [113–121], including hair follicles [122]. Patients receiving radiation therapy for various cancers often experience localized hair graying in the irradiated areas. Clinical records and case studies documented the incidence and severity of radiation-induced hair graying [102, 123], providing valuable insights into the effects of radiation on hair pigmentation.

Patients undergoing radiation therapy for head and neck cancers, brain tumors, or other malignancies in close proximity to the scalp frequently experience localized hair graying within the radiation field. The severity and extent of hair graying can vary depending on the radiation dose, fractionation, and treatment duration. Clinical observations reveal that the onset and progression of radiation-induced hair graying can occur over different timeframes. In some cases, rapid hair graying within weeks or months after initiating radiation therapy has been reported. Alternatively, a delayed onset of hair graying may manifest months or even years after the completion of radiation treatment. These variations in timing suggest that the effects of radiation on hair pigmentation may involve complex interactions between acute and chronic cellular responses to radiation exposure as well as the functional state of the hair follicle during exposure. It is important to note that radiation-induced hair graying typically occurs within the irradiated field, leading to distinct patterns of graying based on the radiation treatment site. For example, patients receiving radiation therapy for brain tumors may experience graying in specific regions of the scalp corresponding to the radiation beam entry and exit points. This localized graying provides clinical evidence of the direct impact of radiation on hair follicles in the irradiated area.

Severity of radiation-induced hair graying can also vary among individuals. Factors such as the radiation dose, treatment techniques, individual susceptibility, and genetic predisposition may influence the extent and rapidity of hair graying. Hair graying induced by radiation therapy is often permanent, and the affected hair typically does not regain its original pigmentation even after the completion of treatment.

Beyond the aesthetic concerns, radiation-induced hair graying can have significant psychosocial implications for patients. Changes in physical appearance, particularly those associated with cancer treatments, can impact an individual’s self-image, self-esteem, and quality of life. Hair graying may act as a visual reminder of the patient’s cancer journey, potentially leading to emotional distress, body image issues, and reduced overall well-being. Therefore, the psychological impact of radiation-induced hair graying should not be underestimated, and supportive care measures should be considered for patients experiencing these changes. Clinical observations of hair graying in radiation therapy patients have contributed to our understanding of the effects of radiation on hair pigmentation. These observations highlight the need for further research to elucidate the underlying mechanisms and develop interventions to prevent or mitigate radiation-induced hair graying. By understanding the cellular and molecular processes involved, it is possible to explore strategies to protect hair follicles from radiation damage and potentially preserve hair pigmentation during cancer treatments.

### Experimental models and methodologies

The dominant experimental models for evaluation of hair graying are in vivo mouse models and human hair follicle explants. Research involves visual inspection and quantification of the extent and severity of hair depigmentation. Various assessment methods have been used, including macroscopic observation, digital imaging, and quantitative measurements of hair pigmentation using specialized software. These approaches allow researchers to quantify the percentage of gray or white hairs, assess the rate of hair graying, and compare different experimental groups or treatment conditions. Histological analysis of the hair follicles is also a valuable technique in studying radiation-induced hair graying. It is possible to examine the histological features of the hair follicles, including the number and morphology of melanocytes, melanin content, follicular structure, and the presence of apoptotic or senescent cells. Techniques like immunohistochemistry and in situ hybridization enable the visualization and characterization of specific cellular markers associated with changes in hair pigmentation. Recently, omics analyses including RNA sequencing and LC–MS/MS-based proteomics have elevated our understanding to the level of entire signaling pathways and molecular cascades that are reprogrammed in concert with hair pigmentation changes.

Animal studies conducted throughout the twentieth century provide much of the basis for our current understanding of the molecular and cellular mechanisms involved in radiation-induced hair graying. Experiments using laboratory animals, including mice, hamsters, and non-human primates, exposed to ionizing radiation demonstrated changes in hair pigmentation consistent with human observations [27, 104–107, 109, 110]. These animal models allowed for controlled radiation exposure to dissect the timing, dosage, and frequency required to induce the phenotype [100, 124–127]. These observations provide the foundation for using irradiation-induced hair graying as an experimental model to study the mechanisms underlying this phenomenon and its relationship to aging [80], as well as employing this model to test therapeutic approaches to reverse hair graying [24, 111].

**Fig. 1:**
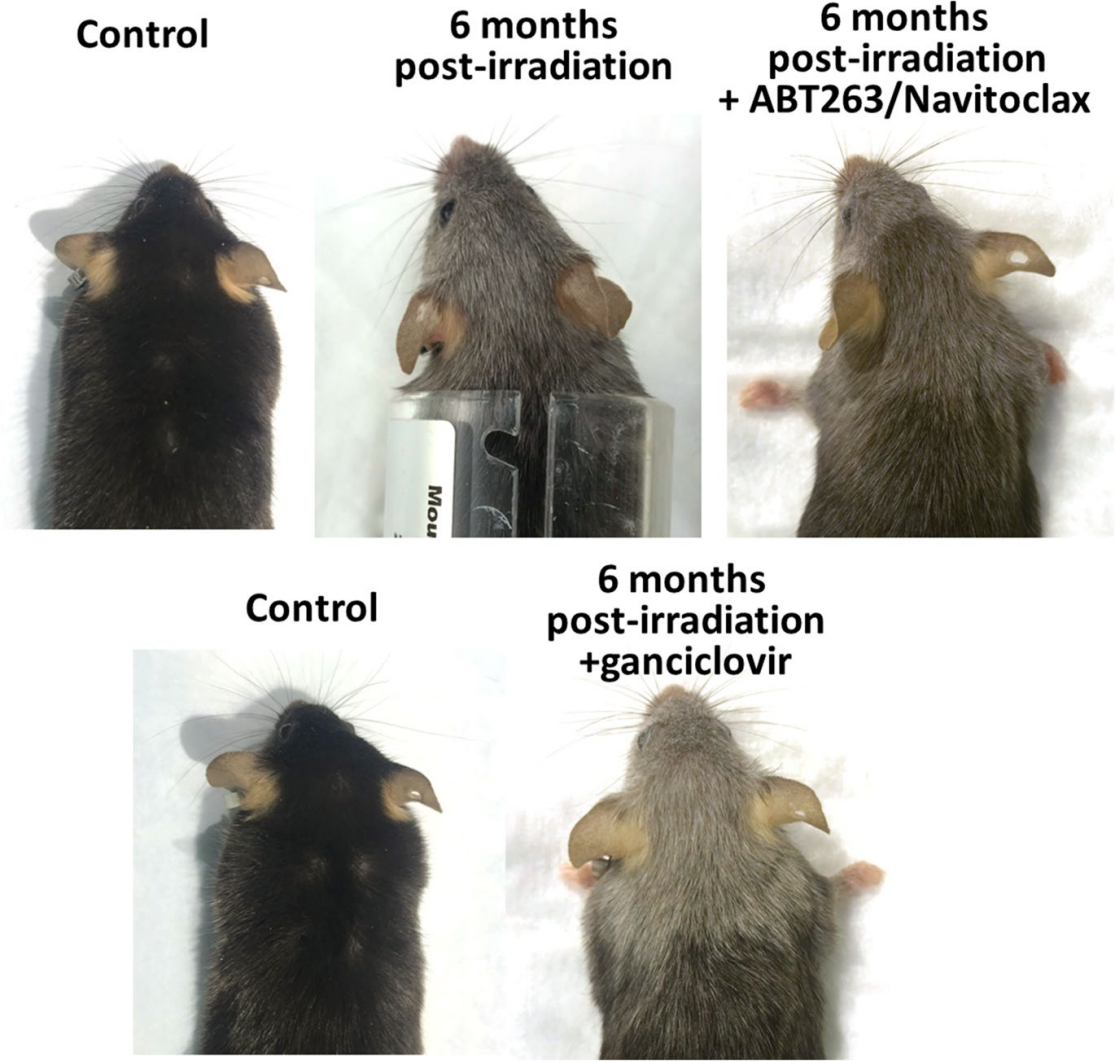
Representative images of γ-irradiated mice with and without senolytic treatment. Male transgenic 3-MR mice (p16-3MR mice [132, 133]) that carry a trimodal fusion protein (3MR) under the control of the p16^*Ink4a*^ promoter were subjected to clinically relevant whole brain irradiation protocol (5 Gy twice weekly for a total cumulative dose of 40 Gy) at 3 months of age [134]. Radiation was administered using a ^137^Cesium gamma irradiator (GammaCell 40, Nordion International). A Cerrobend® shield was utilized to minimize exposure outside the brain. After 3 months, to eliminate senescent cells, one group received the senolytic drug ABT263 (Navitoclax) via oral gavage, 50 mg/kg dissolved in phosal PG:PEG400:ethanol (60:30:10)) [133, 135, 136] for 5 days and for 2 cycles with a 2-week interval between cycles. 3MR contains the herpes simplex virus thymidine kinase, which allows the selective elimination of p16-positive senescent cells by administering the prodrug ganciclovir [137]. Thus, to deplete senescent cells, another group received ganciclovir (i.p. 25 mg/kg/daily in PBS) for 5 days and for 2 cycles with a 2-week interval between cycles. Animals were photographed 3 months after the initiation of senolytic treatment

Mouse models, in particular, have been used extensively to investigate radiation-induced hair graying [107, 108, 110, 128] (Fig. 1). Mice offer several advantages, including their relatively short lifespan, ease of handling, and well-characterized hair follicle cycling. Experimental protocols typically involve exposing the dorsal skin of the mice to a low dose of ionizing irradiation using specialized radiation sources. Sources of ionizing radiation to induce hair graying experimentally include γ-rays from radioactive sources like cesium-137 and X-rays generated from a non-radioactive X-ray tube [129]. In the last two decades, the technology of X-ray irradiators has matured to provide parallel efficiency and improved safety in comparison to radioactive sources, and irradiators using cesium-137 fallen out of favor as regulatory agencies mitigate global security risks associated with easily obtainable radioactive substances [130, 131]. Thus, ~ 5 Gy delivered by an X-ray irradiator is commonly used. Importantly, this dose is sublethal and insufficient to ablate bone marrow, yet effective for inducing gray hair in mouse.

As early as the 1950s, it was acknowledged that radiation-induced graying in mouse depends on irradiation dose and dose rate, type and size of hair (zigzag hairs are most sensitive), and stage of the hair cycle. These early studies also provided the basis for interrogating differences in the DNA damage response between the stem and non-stem components of the regenerative pigmentary tissue system. In mouse, telogen hair follicles are notably more susceptible to IR-induced hair graying in comparison to anagen follicles. Because telogen follicles contain only quiescent melanocyte stem cells and no amplifying or differentiated melanocytes, this suggests heterogeneity in the cellular response to IR and a dependency on cell state (quiescence) and differentiation status (stem). More recently, lineage mapping using a melanocyte lineage–specific reporter gene (DCT-H2B-GFP) and selective depletion of non-quiescent melanocyte stem cells confirmed that it is the quiescent stem cells in the hair bulge that are most highly radiosensitive. In response to IR, quiescent melanocyte stem cells acquire irreparable DNA damage but do not undergo apoptosis. Rather, irradiated stem cells prematurely differentiate without self-renewal [90, 138]. Quiescent cells rely on non-homologous end joining rather than homologous recombination for repairing DNA double-stranded breaks and is speculated to be the reason for quiescent melanocyte stem cell radiosensitivity. The use of genetic mouse models further shows that IR-induced melanocyte stem cell differentiation is not reliant on p53, p19ARF, or p16INK4a and is distinct from senescence. Advanced non-invasive methods to isolate telogen follicles from mouse skin using suction provide additional feasibility for investigating the molecular mechanism that drives stem cell differentiation due to DNA damage [139].

Because of the robust graying induced by IR at telogen, mouse models have been used predominately to dissect the effects of IR on melanocyte stem cells. As a complementary alternative, explants of human anagen hair follicles are an effective model to reveal IR’s impact on melanogenesis and maintenance of terminally differentiated melanocytes. Anagen hair follicles grown ex vivo continue to grow and produce pigmented hair shafts. Follicles respond quickly to IR perturbation, and within 3 days after exposure (5–8 Gy), matrix melanocytes are reduced in number and exhibit reduced melanin content; a small proportion upregulate the DNA damage marker H2AX or the senescent marker P16. Undifferentiated melanocytes within the ORS and proximal hair bulb (presumed melanocyte stem cells) react to IR (5–8 Gy) similarly to that observed in mice by prematurely upregulating the differentiation markers TRP1 and TYR and producing ectopic pigment. These responses altogether mimic those observed in white hair follicles obtained from aged individuals suggesting that irradiation can model aspects of aging in the HFPU [111].

The experimental model of IR-induced hair graying in mice offers several advantages. Dosage and timing of radiation exposure can be precisely controlled, allowing researchers to study dose-dependent effects and to investigate the threshold doses required to induce hair graying and the kinetics of hair graying [140]. It is possible to employ multiple time points post-irradiation to assess the temporal progression of hair graying and monitor the cellular and molecular changes over time. The mouse model also provides researchers with a self-controlled experimental design. By comparing the irradiated areas with non-irradiated control areas in the same animals, researchers can effectively control for individual variations and genetic differences among the subjects minimizing confounding factors and strengthening the ability to attribute observed hair graying specifically to radiation exposure. Further, the IR-induced hair graying model allows for reproducible and standardized protocols, ensuring consistency across different studies.

Animal studies also provide opportunities for intervention and therapeutic exploration [24]. Previous studies have explored the use of antioxidants, anti-inflammatory agents, and other compounds to mitigate the effects of radiation on hair pigmentation. For example, a recent study demonstrated the efficacy of the combination treatment with cyclosporine A and minoxidil, along with RT175, a non-immunosuppressive immunophilin ligand in this model [24]. These studies have shown promising results in preventing or reducing hair graying in irradiated mice, highlighting this model as a potential screening tool to identify novel biological pathways involved in melanocyte stem cell and HFPU rejuvenation in response to genotoxic stress [24]. The ability to genetically manipulate specific genes or signaling pathways in mice further enables our ability to gain mechanistic insight into key players in this process.

The use of in vitro cell culture models has also contributed to our understanding of radiation on melanocyte function. Melanocyte cell lines or primary melanocyte cultures can be irradiated and subsequently assessed for changes in melanin production, cellular senescence markers, and gene expression profiles. These in vitro models allow for controlled experiments and provide insights into the molecular pathways underlying radiation-induced changes in melanocyte function and hair graying.

### Exploring the irradiation (IR) model to investigate the role of cellular senescence in hair graying and graying reversal

While the precise mechanisms through which radiation exposure contributes to hair graying are still being elucidated, the evidence suggests that radiation-induced DNA damage and oxidative stress affecting both melanocyte stem cells and differentiated melanocytes directly play crucial roles and mimics the aging phenotype. However, less attention has been given to the role of the niche as a contributing factor to the decline of the pigmentary system after IR, or in age-related hair graying. Significant evidence implicates the niche in both melanocyte stem cell support and HFPU maintenance. For instance, exogenous growth factors can prevent hair graying caused by repeated depilation [141], and mutations conditionally affecting hair follicle stem cells (i.e., *Col17a1*) can also lead to disruptions in melanocyte stem cell maintenance and cause premature gray hair [142]. In growing human hairs, hair follicle keratinocytes especially those differentiating within the inner root sheath, more so than melanocytes, exhibit markers of sustained oxidative stress and apoptosis potentially creating their own stressful microenvironment as part of natural hair cycling [83].

It is likely that the keratinocyte-melanocyte relationship also plays an important role in the context of IR-induced hair graying. IR slows hair regrowth after depilation and reduces the colony-forming capacity of follicular keratinocyte stem cells. Reconstitution of hairs using irradiated host skin and non-irradiated donor melanocyte stem cells results in gray hair, suggesting irradiated hair follicle keratinocytes are insufficient to support or can act as mediators of melanocyte susceptibility to IR [143]. In reference to the latter, how hair follicle keratinocytes negatively impact melanocyte stem cells or the HFPU in the context of IR is unknown, but one understudied aspect of radiation exposure that has not been directly implicated in hair graying is cellular senescence. Cellular senescence is a state of irreversible growth arrest characterized by altered cellular function and the secretion of pro-inflammatory molecules known as the senescence-associated secretory phenotype (SASP) [144–149]. The interplay between cellular senescence and hair graying likely involves complex molecular pathways that include cells of the melanocyte lineage along with senescing cells within the niche.

In several contexts, cellular senescence is an intrinsic aspect of melanocyte pathology, particularly in vitiligo and melanoma [111–114]. Senescent epidermal melanocytes have also been implicated in driving skin aging [150]. However, the contribution of senescent melanocytes to hair graying in response to radiation or aging remains unclear. In mice, the dominant response by melanocyte stem cells in response to IR is not senescence. Rather, melanocyte stem cells prematurely differentiate, and this process occurs even in mice that do not express P16. Differentiating/ed melanocytes in anagen hair bulbs of mice are radioresistant, and exposure to IR does not induce significant hair graying in the time frame in which pigmented hair shafts are produced [90]. In human scalp hairs, on the other hand, senescent hair matrix melanocytes are observed with both IR and aging, suggesting that this is a characteristic unique to human hair bulb melanocytes and is perhaps attributable to the much longer time period these cells remain functional during the lengthy anagen hair growth period in humans [111]. Outside of melanocytes, and in both mouse and human, radiation exposure induces robust cellular senescence within the hair follicle similar to that observed in aged hair follicles [111, 139]. Because SASP molecules secreted by senescent cells can influence neighboring cells, including melanocytes, senescent keratinocytes may contribute to the chronic inflammation observed in hair follicles and further influence the hair-graying process. In mice, although the initial insult that causes hair graying does not depend directly on senescent melanocyte stem cells or melanocytes, repeated depilation shows that irradiated mice continue to gray across multiple hair cycles, and whether senescent cells participate in this progressive process is unknown. Cellular senescence is recognized as a fundamental process in aging and age-related diseases [144–149]. Understanding the role of cellular senescence in hair graying, including its potential induction by radiation exposure, provides valuable insights into the underlying mechanisms of how senescence augments the decline of regenerative tissues.

Exposure to ionizing radiation can induce cellular senescence through various mechanisms. DNA damage resulting from radiation exposure can activate cellular stress response pathways, such as the DNA damage response (DDR) pathway [151]. DDR signaling triggers cell cycle arrest and can lead to the induction of cellular senescence [152, 153]. Strategies aimed at preventing DNA damage, modulating cellular senescence or the SASP, and eliminating senescent cells (senolytic treatments) may offer promising avenues for future research and the development of interventions to prevent premature development of age-related pathologies [154–157] including hair graying. As an example, Fig. 1 depicts γ-irradiated mice in two different senolytic models, administration of ganciclovir to p16-3MR mice [132, 133], and administration of ABT/Navitoclax to wild-type mice. In this study, the primary focus was to investigate the impact of whole-brain irradiation on cerebrovascular outcomes [133]. However, changes in hair color were also recorded as a secondary outcome during the course of the study. These preliminary data showed that systemic senolytic treatment initiated 3 months post-irradiation did not reverse or curtail hair graying in irradiated mice (Fig. 1). This suggests that senescence, be it intrinsic or extrinsic to the melanocyte lineage, may not contribute to the continued hair graying observed in irradiated mice over time, but additional studies should be performed to solidify this finding. When using the mouse model, understanding the dynamics of hair cycling and hair cycle stage during irradiation and treatment is critical to interpreting the experimental outcomes. Repeated depilation in mice has the added benefit of addressing senescence chronologically in the same animals and allows for direct assessment of stem cell regenerative potential. While the mouse model is fantastic for investigating the effects of perturbation or therapeutics on melanocyte stem cell function, it is limited in its ability to investigate long-term effects of IR or age on differentiated hair follicle melanocytes. Thus, expanding studies of senolytics to explants of human anagen hair follicles serves as an important and complementary approach if we want to understand senolytics in the context of human hair graying. It is also important to note that various senolytic regimens may have side effects that can confound the results. For example, previous studies showed that defective self-maintenance of melanocyte stem cells promotes hair graying, and this process is accelerated dramatically with Bcl2 deficiency, which causes selective apoptosis of melanocyte stem cells [27]. In the field of geroscience, Bcl2 inhibitors (e.g., ABT263/Navitoclax) are often used as senolytic treatments [133, 135, 154, 155]. However, administration of ABT263 to mice results in regional hair graying at the site of the injection (Ungvari and coworkers, unpublished observation, 2021), probably due to the aforementioned side effects of the drug. Thus, future studies should employ specific senolytic treatments without side effects that potentially impact hair color.

## Future Directions and Conclusions

In conclusion, IR-induced hair graying serves as a valuable experimental model to study the intricate processes underlying hair graying, with a particular focus on the potential role of radiation-induced oxidative stress, DNA damage, and cellular senescence. By unraveling the molecular pathways involved, this research contributes to our broader understanding of hair graying in aging and sheds light on potential therapeutic targets for preventing or reversing this aesthetic change. These studies can also answer basic biological questions regarding the role of the niche and cellular senescence in tissue regeneration in response to genotoxic stress and delineate short- and long-term effects of radiation on stem and differentiated cell function. Further investigations in this field, using a combination of both mouse and human models, are warranted to unlock the full potential of this experimental model and translate the findings into clinical applications.

Long-term studies exploring the reversibility of radiation-induced hair graying with novel anti-aging interventions, including specific senolytic treatments, are also warranted. In humans, repigmentation of gray or white hair is a well-documented phenomenon and can occur both naturally along an individual hair shaft or in response to chemicals and medications [22]. One dramatic example includes complete gray hair reversal as a side effect of cancer therapy (e.g., anti-programmed cell death 1 (anti-PD-1) and anti-programmed cell death ligand 1 (anti-PD-L1) therapy for lung cancer [23]). Preclinical models of hair graying, like the IR-induced models described above, can help elucidate mechanisms by which hair repigmentation occurs. This may include mechanisms that directly mitigate the effects of irradiation (e.g., enhancing antioxidant levels) but can also include gain-of-function mechanisms unrelated to the initial insult (e.g., promoting repopulation of the hair bulb from inactive melanocyte stem cells). The application of multiomics technologies is anticipated to offer novel insights into the biology of hair aging, ultimately aiding in the identification of specific targets for the development of effective therapeutic interventions [158]. A recent study found that caloric restriction, an anti-aging dietary regimen, significantly prolongs the time it takes for hair to turn gray in response to IR-induced DNA damage in mice [159]. The research revealed that caloric restriction extends the resting phase of hair follicles by keeping hair follicle stem cells in a quiescent state, which temporarily prevents the depletion of melanoblasts and delays hair graying [159]. These promising preclinical findings underscore the need for further investigation into the effects of anti-aging dietary regimens, potentially in combination with other treatments, in human subjects. Beyond identifying therapeutic targets for preventing or treating hair graying in both radiation-exposed individuals and the general aging population for the purpose of aesthetics, the melanocyte stem cell, HFPU, and IR-induced hair graying provide a powerful tool to investigate and test mechanisms associated with regenerative tissue decline with aging and the effects of pro-longevity interventions during the progression of stem cell differentiation.

## References

[1] Steingrimsson E, Copeland NG, Jenkins NA (2005). Melanocyte stem cell maintenance and hair graying. Cell.

[2] Jo SK, Lee JY, Lee Y, Kim CD, Lee JH, Lee YH (2018). Three streams for the mechanism of hair graying. Ann Dermatol.

[3] Tobin DJ, Paus R (2001). Graying: gerontobiology of the hair follicle pigmentary unit. Exp Gerontol.

[4] Schouwey K, Beermann F (2008). The Notch pathway: hair graying and pigment cell homeostasis. Histol Histopathol.

[5] Mirmirani P (2015). Age-related hair changes in men: mechanisms and management of alopecia and graying. Maturitas.

[6] Panhard S, Lozano I, Loussouarn G (2012). Greying of the human hair: a worldwide survey, revisiting the ‘50’ rule of thumb. Br J Dermatol.

[7] Mahendiratta S, Sarma P, Kaur H, Kaur S, Kaur H, Bansal S, Prasad D, Prajapat M, Upadhay S, Kumar S, Kumar H, Singh R, Singh A, Mishra A, Prakash A, Medhi B (2020). Premature graying of hair: risk factors, co-morbid conditions, pharmacotherapy and reversal-a systematic review and meta-analysis. Dermatol Ther.

[8] Shin H, Ryu HH, Yoon J, Jo S, Jang S, Choi M, Kwon O, Jo SJ (2015). Association of premature hair graying with family history, smoking, and obesity: a cross-sectional study. J Am Acad Dermatol.

[9] Sharma N, Dogra D (2018). Association of epidemiological and biochemical factors with premature graying of hair: a case-control study. Int J Trichology.

[10] Thompson KG, Marchitto MC, Ly BCK, Chien AL (2019). Evaluation of physiological, psychological, and lifestyle factors associated with premature hair graying. Int J Trichology.

[11] Mendelsohn AR, Larrick JW (2020). The danger of being too sympathetic: norepinephrine in Alzheimer’s Disease and Graying of Hair. Rejuvenation Res.

[12] Zhang B, He M, Rachmin I, Yu X, Kim S, Fisher DE, Hsu YC (2021). Melanocortin 1 receptor is dispensable for acute stress induced hair graying in mice. Exp Dermatol.

[13] Clarke, L. H. Women, aging, and beauty culture: navigating the social perils of looking old. (2017). https://www.ingentaconnect.com/contentone/asag/gen/2018/00000041/00000004/art00016. Accessed 09/01/2023.

[14] Hofmeier SM, Runfola CD, Sala M, Gagne DA, Brownley KA, Bulik CM (2017). Body image, aging, and identity in women over 50: the gender and body image (GABI) study. J Women Aging.

[15] Synnott A (1987). Shame and glory: a sociology of hair. Br J Sociol.

[16] Ward R, Holland C (2011). ‘If I look old, I will be treated old’: hair and later-life image dilemmas. Ageing Soc.

[17] Bian Y, Wei G, Song X, Yuan L, Chen H, Ni T, Lu D (2019). Global downregulation of pigmentation-associated genes in human premature hair graying. Exp Ther Med.

[18] Acer E, Kaya Erdogan H, Kocaturk E, Saracoglu ZN, Alatas O, Bilgin M (2020). Evaluation of oxidative stress and psychoemotional status in premature hair graying. J Cosmet Dermatol.

[19] Kondo H, Lane MA, Yonezawa Y, Ingram DK, Cutler RG, Roth GS (1995). Effects of aging and dietary restriction on activity of monkey serum in promoting fibroblast migration. Mech Ageing Dev.

[20] Tang D, Wu J, Wang Y, Cui H, Tao Z, Lei L, Zhou Z, Tao S (2022). Dietary restriction attenuates inflammation and protects mouse skin from high-dose ultraviolet B irradiation. Rejuvenation Res.

[21] Forni MF, Peloggia J, Braga TT, Chinchilla JEO, Shinohara J, Navas CA, Camara NOS, Kowaltowski AJ (2017). Caloric restriction promotes structural and metabolic changes in the skin. Cell Rep.

[22] Yale K, Juhasz M, Atanaskova MN (2020). Medication-induced repigmentation of gray hair: a systematic review. Skin Appendage Disord.

[23] Rivera N, Boada A, Bielsa MI, Fernandez-Figueras MT, Carcereny E, Moran MT, Ferrandiz C (2017). Hair repigmentation during immunotherapy treatment with an anti-programmed cell death 1 and anti-programmed cell death ligand 1 agent for lung cancer. JAMA Dermatol.

[24] Anderson ZT, Palmer JW, Idris MI, Villavicencio KM, Le G, Cowart J, Weinstein DE, Harris ML (2021). Topical RT1640 treatment effectively reverses gray hair and stem cell loss in a mouse model of radiation-induced canities. Pigment Cell Melanoma Res.

[25] D'Mello SA, Finlay GJ, Baguley BC, Askarian-Amiri ME (2016). Signaling pathways in melanogenesis. Int J Mol Sci..

[26] Ohbayashi N, Fukuda M. Recent advances in understanding the molecular basis of melanogenesis in melanocytes. F1000Res. 2020;9:F1000 Faculty Rev-608.

[27] Nishimura EK, Granter SR, Fisher DE (2005). Mechanisms of hair graying: incomplete melanocyte stem cell maintenance in the niche. Science.

[28] Choi YJ, Yoon TJ, Lee YH (2008). Changing expression of the genes related to human hair graying. Eur J Dermatol.

[29] Zhou S, Zeng H, Huang J, Lei L, Tong X, Li S, Zhou Y, Guo H, Khan M, Luo L, Xiao R, Chen J, Zeng Q (2021). Epigenetic regulation of melanogenesis. Ageing Res Rev.

[30] Raposo G, Marks MS (2007). Melanosomes–dark organelles enlighten endosomal membrane transport. Nat Rev Mol Cell Biol.

[31] Bellono NW, Escobar IE, Lefkovith AJ, Marks MS, Oancea E (2014). An intracellular anion channel critical for pigmentation. Elife.

[32] Chen J, Zheng Y, Hu C, Jin X, Chen X, Xiao Y, Wang C (2022). Hair graying regulators beyond hair follicle. Front Physiol.

[33] Kageyama T, Shimizu A, Anakama R, Nakajima R, Suzuki K, Okubo Y, Fukuda J. Reprogramming of three-dimensional microenvironments for in vitro hair follicle induction. Sci Adv. 2022;8:eadd4603. 10.1126/sciadv.add4603 PMC9586475 36269827

[34] Sun Q, Lee W, Hu H, Ogawa T, De Leon S, Katehis I, Lim CH, Takeo M, Cammer M, Taketo MM, Gay DL, Millar SE, Ito M (2023). Dedifferentiation maintains melanocyte stem cells in a dynamic niche. Nature.

[35] Zheng Q, Zhang X, Bao P, Zhou X, Chu M, Guo X, Liang C, Pan H, Yan P (2022). Understanding mammalian hair follicle ecosystems by single-cell RNA sequencing. Animals (Basel)..

[36] Zheng W, Xu CH (2023). Innovative approaches and advances for hair follicle regeneration. ACS Biomater Sci Eng.

[37] Kadekaro AL, Kanto H, Kavanagh R, Abdel-Malek Z (2003). Significance of the melanocortin 1 receptor in regulating human melanocyte pigmentation, proliferation, and survival. Ann N Y Acad Sci.

[38] Kauser S, Thody AJ, Schallreuter KU, Gummer CL, Tobin DJ (2005). A fully functional proopiomelanocortin/melanocortin-1 receptor system regulates the differentiation of human scalp hair follicle melanocytes. Endocrinology.

[39] Sturm RA, Teasdale RD, Box NF (2001). Human pigmentation genes: identification, structure and consequences of polymorphic variation. Gene.

[40] Botchkareva NV, Khlgatian M, Longley BJ, Botchkarev VA, Gilchrest BA (2001). SCF/c-kit signaling is required for cyclic regeneration of the hair pigmentation unit. FASEB J.

[41] Rabbani P, Takeo M, Chou W, Myung P, Bosenberg M, Chin L, Taketo MM, Ito M (2011). Coordinated activation of Wnt in epithelial and melanocyte stem cells initiates pigmented hair regeneration. Cell.

[42] Liao CP, Booker RC, Morrison SJ, Le LQ (2017). Identification of hair shaft progenitors that create a niche for hair pigmentation. Genes Dev.

[43] Takeo M, Lee W, Rabbani P, Sun Q, Hu H, Lim CH, Manga P, Ito M (2016). EdnrB governs regenerative response of melanocyte stem cells by crosstalk with Wnt signaling. Cell Rep.

[44] Pss R, Madhunapantula SV, Betkerur JB, Bovilla VR, Shastry V (2022). Melanogenesis markers expression in premature graying of hair: a cross-sectional study. Skin Pharmacol Physiol.

[45] Nishimura EK, Jordan SA, Oshima H, Yoshida H, Osawa M, Moriyama M, Jackson IJ, Barrandon Y, Miyachi Y, Nishikawa S (2002). Dominant role of the niche in melanocyte stem-cell fate determination. Nature.

[46] Horikawa T, Norris DA, Johnson TW, Zekman T, Dunscomb N, Bennion SD, Jackson RL, Morelli JG (1996). DOPA-negative melanocytes in the outer root sheath of human hair follicles express premelanosomal antigens but not a melanosomal antigen or the melanosome-associated glycoproteins tyrosinase, TRP-1, and TRP-2. J Invest Dermatol.

[47] Takada K, Sugiyama K, Yamamoto I, Oba K, Takeuchi T (1992). Presence of amelanotic melanocytes within the outer root sheath in senile white hair. J Invest Dermatol.

[48] Slominski A, Paus R, Plonka P, Chakraborty A, Maurer M, Pruski D, Lukiewicz S (1994). Melanogenesis during the anagen-catagen-telogen transformation of the murine hair cycle. J Invest Dermatol.

[49] Tobin DJ, Hagen E, Botchkarev VA, Paus R (1998). Do hair bulb melanocytes undergo apoptosis during hair follicle regression (catagen)?. J Invest Dermatol.

[50] Geyfman M, Plikus MV, Treffeisen E, Andersen B, Paus R (2015). Resting no more: re-defining telogen, the maintenance stage of the hair growth cycle. Biol Rev Camb Philos Soc.

[51] Silver AF, Chase HB, Potten CS (1969). Melanocyte precursor cells in the hair follicle germ during the dormat stage (telogen). Experientia.

[52] Courtois M, Loussouarn G, Hourseau C, Grollier JF (1995). Ageing and hair cycles. Br J Dermatol.

[53] Plikus MV, Widelitz RB, Maxson R, Chuong CM (2009). Analyses of regenerative wave patterns in adult hair follicle populations reveal macro-environmental regulation of stem cell activity. Int J Dev Biol.

[54] Harris ML, Buac K, Shakhova O, Hakami RM, Wegner M, Sommer L, Pavan WJ (2013). A dual role for SOX10 in the maintenance of the postnatal melanocyte lineage and the differentiation of melanocyte stem cell progenitors. PLoS Genet.

[55] Arck PC, Overall R, Spatz K, Liezman C, Handjiski B, Klapp BF, Birch-Machin MA, Peters EM (2006). Towards a “free radical theory of graying”: melanocyte apoptosis in the aging human hair follicle is an indicator of oxidative stress induced tissue damage. FASEB J.

[56] Wang S, Kang Y, Qi F, Jin H (2023). Genetics of hair graying with age. Ageing Res Rev.

[57] Lyu Y, Ge Y (2022). Toward elucidating epigenetic and metabolic regulation of stem cell lineage plasticity in skin aging. Front Cell Dev Biol.

[58] Pospiech E, Kukla-Bartoszek M, Karlowska-Pik J, Zielinski P, Wozniak A, Boron M, Dabrowski M, Zubanska M, Jarosz A, Grzybowski T, Ploski R, Spolnicka M, Branicki W (2020). Exploring the possibility of predicting human head hair greying from DNA using whole-exome and targeted NGS data. BMC Genomics.

[59] Wilson MM, Danielian PS, Salus G, Ferretti R, Whittaker CA, Lees JA (2023). BMI1 is required for melanocyte stem cell maintenance and hair pigmentation. Pigment Cell Melanoma Res..

[60] Fialkowski AC, Levy DJ, Watkins-Chow DE, Palmer JW, Darji R, Tiwari HK, Pavan WJ, Harris ML. Identification of gene variants associated with melanocyte stem cell differentiation in mice predisposed for hair graying. G3 (Bethesda). 2019;9(3):817–827. 10.1534/g3.118.200965 PMC6404613 30651286

[61] Harris ML, Levy DJ, Watkins-Chow DE, Pavan WJ (2015). Ectopic differentiation of melanocyte stem cells is influenced by genetic background. Pigment Cell Melanoma Res.

[62] Harris ML, Pavan WJ (2013). Postnatal lineage mapping of follicular melanocytes with the Tyr::CreER(T) (2) transgene. Pigment Cell Melanoma Res.

[63] Harris ML, Fufa TD, Palmer JW, Joshi SS, Larson DM, Incao A, Gildea DE, Trivedi NS, Lee AN, Day CP, Michael HT, Hornyak TJ, Merlino G, Program NCS, Pavan WJ (2018). A direct link between MITF, innate immunity, and hair graying. PLoS Biol.

[64] Pavan WJ, Sturm RA (2019). The genetics of human skin and hair pigmentation. Annu Rev Genomics Hum Genet.

[65] Rees JL, Harding RM (2012). Understanding the evolution of human pigmentation: recent contributions from population genetics. J Invest Dermatol.

[66] Sturm RA (2009). Molecular genetics of human pigmentation diversity. Hum Mol Genet.

[67] Menasche G, Ho CH, Sanal O, Feldmann J, Tezcan I, Ersoy F, Houdusse A, Fischer A, de Saint BG (2003). Griscelli syndrome restricted to hypopigmentation results from a melanophilin defect (GS3) or a MYO5A F-exon deletion (GS1). J Clin Invest.

[68] Pastural E, Barrat FJ, Dufourcq-Lagelouse R, Certain S, Sanal O, Jabado N, Seger R, Griscelli C, Fischer A, de Saint BG (1997). Griscelli disease maps to chromosome 15q21 and is associated with mutations in the myosin-Va gene. Nat Genet.

[69] Westbroek W, Tuchman M, Tinloy B, De Wever O, Vilboux T, Hertz JM, Hasle H, Heilmann C, Helip-Wooley A, Kleta R, Gahl WA (2008). A novel missense mutation (G43S) in the switch I region of Rab27A causing Griscelli syndrome. Mol Genet Metab.

[70] Lang D, Lu MM, Huang L, Engleka KA, Zhang M, Chu EY, Lipner S, Skoultchi A, Millar SE, Epstein JA (2005). Pax3 functions at a nodal point in melanocyte stem cell differentiation. Nature.

[71] Adhikari K, Fontanil T, Cal S, Mendoza-Revilla J, Fuentes-Guajardo M, Chacon-Duque JC, Al-Saadi F, Johansson JA, Quinto-Sanchez M, Acuna-Alonzo V, Jaramillo C, Arias W, Barquera Lozano R, Macin Perez G, Gomez-Valdes J, Villamil-Ramirez H, Hunemeier T, Ramallo V, Silva de Cerqueira CC, Hurtado M, Villegas V, Granja V, Gallo C, Poletti G, Schuler-Faccini L, Salzano FM, Bortolini MC, Canizales-Quinteros S, Rothhammer F, Bedoya G, Gonzalez-Jose R, Headon D, Lopez-Otin C, Tobin DJ, Balding D and Ruiz-Linares A. A genome-wide association scan in admixed Latin Americans identifies loci influencing facial and scalp hair features. Nat Commun. 2016;7:10815. 10.1038/ncomms10815 PMC4773514 26926045

[72] Praetorius C, Grill C, Stacey SN, Metcalf AM, Gorkin DU, Robinson KC, Van Otterloo E, Kim RS, Bergsteinsdottir K, Ogmundsdottir MH, Magnusdottir E, Mishra PJ, Davis SR, Guo T, Zaidi MR, Helgason AS, Sigurdsson MI, Meltzer PS, Merlino G, Petit V, Larue L, Loftus SK, Adams DR, Sobhiafshar U, Emre NC, Pavan WJ, Cornell R, Smith AG, McCallion AS, Fisher DE, Stefansson K, Sturm RA, Steingrimsson E (2013). A polymorphism in IRF4 affects human pigmentation through a tyrosinase-dependent MITF/TFAP2A pathway. Cell.

[73] Sen P, Shah PP, Nativio R, Berger SL (2016). Epigenetic mechanisms of longevity and aging. Cell.

[74] Zhang B, Ma S, Rachmin I, He M, Baral P, Choi S, Goncalves WA, Shwartz Y, Fast EM, Su Y, Zon LI, Regev A, Buenrostro JD, Cunha TM, Chiu IM, Fisher DE, Hsu YC (2020). Hyperactivation of sympathetic nerves drives depletion of melanocyte stem cells. Nature.

[75] Rosenberg AM, Rausser S, Ren J, Mosharov EV, Sturm G, Ogden RT, Patel P, Kumar Soni R, Lacefield C, Tobin DJ, Paus R, Picard M. Quantitative mapping of human hair greying and reversal in relation to life stress. Elife. 2021;10:e67437. 10.7554/eLife.67437 PMC8219384 34155974

[76] Hann SK, Lee HJ (1996). Segmental vitiligo: clinical findings in 208 patients. J Am Acad Dermatol.

[77] Scholtz JR, Williamson C (1951). Vitiligo in apparent dermatomal distribution. AMA Arch Derm Syphilol.

[78] Anderson ZT, Mehl J, Corder KM, Dobrunz LE, Harris ML (2021). A novel mouse model to evaluate neuropeptide Y-mediated melanocyte pathology. Exp Dermatol.

[79] O'Sullivan JDB, Peters EMJ, Amer Y, Atuluru P, Cheret J, Rosenberg AM, Picard M, Paus R (2022). The impact of perceived stress on the hair follicle: towards solving a psychoneuroendocrine and neuroimmunological puzzle. Front Neuroendocrinol.

[80] Paus R (2011). A neuroendocrinological perspective on human hair follicle pigmentation. Pigment Cell Melanoma Res.

[81] Seiberg M (2013). Age-induced hair greying - the multiple effects of oxidative stress. Int J Cosmet Sci.

[82] Saxena S, Gautam RK, Gupta A, Chitkara A (2020). Evaluation of systemic oxidative stress in patients with premature canities and correlation of severity of hair graying with the degree of redox imbalance. Int J Trichology.

[83] Sikkink SK, Mine S, Freis O, Danoux L, Tobin DJ (2020). Stress-sensing in the human greying hair follicle: ataxia telangiectasia mutated (ATM) depletion in hair bulb melanocytes in canities-prone scalp. Sci Rep.

[84] Wood JM, Decker H, Hartmann H, Chavan B, Rokos H, Spencer JD, Hasse S, Thornton MJ, Shalbaf M, Paus R, Schallreuter KU (2009). Senile hair graying: H2O2-mediated oxidative stress affects human hair color by blunting methionine sulfoxide repair. FASEB J.

[85] Shi Y, Luo LF, Liu XM, Zhou Q, Xu SZ, Lei TC (2014). Premature graying as a consequence of compromised antioxidant activity in hair bulb melanocytes and their precursors. PLoS ONE.

[86] Denat L, Kadekaro AL, Marrot L, Leachman SA, Abdel-Malek ZA (2014). Melanocytes as instigators and victims of oxidative stress. J Invest Dermatol.

[87] Hubbard-Smith K, Hill HZ, Hill GJ (1992). Melanin both causes and prevents oxidative base damage in DNA: quantification by anti-thymine glycol antibody. Radiat Res.

[88] Swalwell H, Latimer J, Haywood RM, Birch-Machin MA (2012). Investigating the role of melanin in UVA/UVB- and hydrogen peroxide-induced cellular and mitochondrial ROS production and mitochondrial DNA damage in human melanoma cells. Free Radic Biol Med.

[89] Cohen LE, Tanner DJ, Schaefer HG, Levis WR (1984). Common and uncommon cutaneous findings in patients with ataxia-telangiectasia. J Am Acad Dermatol.

[90] Inomata K, Aoto T, Binh NT, Okamoto N, Tanimura S, Wakayama T, Iseki S, Hara E, Masunaga T, Shimizu H, Nishimura EK (2009). Genotoxic stress abrogates renewal of melanocyte stem cells by triggering their differentiation. Cell.

[91] Yu M, Bell RH, Ho MM, Leung G, Haegert A, Carr N, Shapiro J, McElwee KJ (2012). Deficiency in nucleotide excision repair family gene activity, especially ERCC3, is associated with non-pigmented hair fiber growth. PLoS ONE.

[92] Luo J (2010). WRN protein and Werner syndrome. N Am J Med Sci (Boston).

[93] Kolesar JE, Safdar A, Abadi A, MacNeil LG, Crane JD, Tarnopolsky MA, Kaufman BA (2014). Defects in mitochondrial DNA replication and oxidative damage in muscle of mtDNA mutator mice. Free Radic Biol Med.

[94] Wang S, Jacquemyn J, Murru S, Martinelli P, Barth E, Langer T, Niessen CM, Rugarli EI (2016). The mitochondrial m-AAA protease prevents demyelination and hair greying. PLoS Genet.

[95] Zhang Z, Gong J, Sviderskaya EV, Wei A, Li W. Mitochondrial NCKX5 regulates melanosomal biogenesis and pigment production. J Cell Sci. 2019;132. 10.1242/jcs.232009 PMC6679581 31201282

[96] Singh B, Schoeb TR, Bajpai P, Slominski A, Singh KK (2018). Reversing wrinkled skin and hair loss in mice by restoring mitochondrial function. Cell Death Dis.

[97] Kujoth GC, Hiona A, Pugh TD, Someya S, Panzer K, Wohlgemuth SE, Hofer T, Seo AY, Sullivan R, Jobling WA, Morrow JD, Van Remmen H, Sedivy JM, Yamasoba T, Tanokura M, Weindruch R, Leeuwenburgh C, Prolla TA (2005). Mitochondrial DNA mutations, oxidative stress, and apoptosis in mammalian aging. Science.

[98] Bratic I, Trifunovic A (2010). Mitochondrial energy metabolism and ageing. Biochim Biophys Acta.

[99] Jadkauskaite L, Coulombe PA, Schafer M, Dinkova-Kostova AT, Paus R, Haslam IS. Oxidative stress management in the hair follicle: could targeting NRF2 counter age-related hair disorders and beyond? Bioessays. 2017;39(8). 10.1002/bies.201700029 28685843

[100] Rojo de la Vega M, Zhang DD, Wondrak GT. Topical bixin confers NRF2-dependent protection against photodamage and hair graying in mouse skin. Front Pharmacol. 2018;9:287. 10.3389/fphar.2018.00287 PMC5880955 29636694

[101] Chase HB (1951). Number of entities inactivated by X-rays in graying of hair. Science.

[102] Zeligman I (1952). Graying of hair following epilating doses of x-rays. AMA Arch Derm Syphilol.

[103] Hollingsworth JW, Ishii G, Conard RA (1961). Skin aging and hair graying in Hiroshima. Geriatrics.

[104] Chase HB (1946). Greying induced by x-rays in the mouse. Genetics.

[105] Chase HB (1948). Time-factor with respect to X-ray induced greying in the mouse. Genetics.

[106] Chase HB (1949). Greying of hair; effects produced by single doses of X-rays on mice. J Morphol.

[107] Chase HB, Rauch H. Greying of hair. II. Response of individual hairs in mice to variations in x-radiation. J Morphol. 1950;87:381–91. 10.1002/jmor.1050870209 24538946

[108] Boyland E, Sargent S (1951). The local greying of hair in mice treated with x rays and radiomimetic drugs. Br J Cancer.

[109] Garcia H, Shubik P (1971). Epilation and hair greying in hamsters following one single application of beta rays. J Invest Dermatol.

[110] Burlin TE, Challoner AV, Hutton WC, Magnus IA, Ranu HS, Spittle M (1977). Effects of radiation on the visual appearance and mechanical properties of mouse skin. Br J Radiol.

[111] Dai DM, He Y, Guan Q, Fan ZX, Zhu Y, Wang J, Wu S, Chen J, Le D, Hu ZQ, Qu Q, Miao Y (2023). Modeling human gray hair by irradiation as a valuable tool to study aspects of tissue aging. Geroscience.

[112] Hance RT, Murphy JB. Studies on X-ray effects : XV. the prevention of pigment formation in the hair follicles of colored mice with high voltage X-ray. J Exp Med. 1926;44:339–342. 10.1084/jem.44.3.339 PMC2131773 19869188

[113] Ma J, Shi M, Li J, Chen B, Wang H, Li B, Hu J, Cao Y, Fang B, Zhao RC (2007). Senescence-unrelated impediment of osteogenesis from Flk1+ bone marrow mesenchymal stem cells induced by total body irradiation and its contribution to long-term bone and hematopoietic injury. Haematologica.

[114] Marmary Y, Adar R, Gaska S, Wygoda A, Maly A, Cohen J, Eliashar R, Mizrachi L, Orfaig-Geva C, Baum BJ, Rose-John S, Galun E, Axelrod JH (2016). Radiation-induced loss of salivary gland function is driven by cellular senescence and prevented by IL6 modulation. Cancer Res.

[115] Turnquist C, Beck JA, Horikawa I, Obiorah IE, Von Muhlinen N, Vojtesek B, Lane DP, Grunseich C, Chahine JJ, Ames HM, Smart DD, Harris BT, Harris CC (2019). Radiation-induced astrocyte senescence is rescued by Delta133p53. Neuro Oncol.

[116] Brown PD, Jaeckle K, Ballman KV, Farace E, Cerhan JH, Anderson SK, Carrero XW, Barker FG, Deming R, Burri SH, Menard C, Chung C, Stieber VW, Pollock BE, Galanis E, Buckner JC, Asher AL (2016). Effect of radiosurgery alone vs radiosurgery with whole brain radiation therapy on cognitive function in patients with 1 to 3 brain metastases: a randomized clinical trial. JAMA.

[117] Butler RW, Haser JK (2006). Neurocognitive effects of treatment for childhood cancer. Ment Retard Dev Disabil Res Rev.

[118] Chang EL, Wefel JS, Hess KR, Allen PK, Lang FF, Kornguth DG, Arbuckle RB, Swint JM, Shiu AS, Maor MH, Meyers CA (2009). Neurocognition in patients with brain metastases treated with radiosurgery or radiosurgery plus whole-brain irradiation: a randomised controlled trial. Lancet Oncol.

[119] Conill C, Berenguer J, Vargas M, Lopez-Soriano A, Valduvieco I, Marruecos J, Vilella R (2007). Incidence of radiation-induced leukoencephalopathy after whole brain radiotherapy in patients with brain metastases. Clin Transl Oncol.

[120] DeAngelis LM, Delattre JY, Posner JB (1989). Radiation-induced dementia in patients cured of brain metastases. Neurology.

[121] Freeman CR, Bourgouin PM, Sanford RA, Cohen ME, Friedman HS, Kun LE. Long term survivors of childhood brain stem gliomas treated with hyperfractionated radiotherapy. Clinical characteristics and treatment related toxicities. The Pediatric Oncology Group. Cancer. 1996;77:555–62. 10.1002/(SICI)1097-0142(19960201)77:3<555::AID-CNCR19>3.0.CO;2-3 8630965

[122] Lin SJ, Yue Z, Paus R (2023). Clinical pathobiology of radiotherapy-induced alopecia: a guide toward more effective prevention and hair follicle repair. J Invest Dermatol..

[123] Suntsov AG (1963). Growth of gray hairs after roentgen therapy of dermatomycoses of the scalp. Vestn Dermatol Venerol.

[124] Down JD, Berman AJ, Warhol M, Van Dijken PJ, Ferrara JL, Yeap B, Hellman S, Mauch PM (1989). Late tissue-specific toxicity of total body irradiation and busulfan in a murine bone marrow transplant model. Int J Radiat Oncol Biol Phys.

[125] Spittle MF, Ranu HS, Hutton WC, Challoner AV, Burlin TE (1980). A comparison of different treatment regimes on the visual appearance and mechanical properties of mouse skin. Br J Radiol.

[126] Waldow SM, Lustig RA, Brass-Marlow EL, Nunno MP, Holst RJ, Wallner PE (1990). Effect of Fluosol-DA 20% and oxygen on response of C57BL/6 mice to whole-body irradiation. Int J Radiat Oncol Biol Phys.

[127] Taguchi N, Kitai R, Ando T, Nishimura T, Aoki H, Kunisada T (2022). Protective effect of hydroxygenkwanin against hair graying induced by X-ray irradiation and repetitive plucking. JID Innov.

[128] Galbraith DB, Chase HB (1962). Anomalous greying effect in mice with higher doses of x-rays. Science.

[129] Gibson BW, Boles NC, Souroullas GP, Herron AJ, Fraley JK, Schwiebert RS, Sharp JJ, Goodell MA (2015). Comparison of Cesium-137 and X-ray irradiators by using bone marrow transplant reconstitution in C57BL/6J Mice. Comp Med.

[130] United States Nuclear Regulatory Commission. 2005. EA 05–090: licensees authorized to possess radioactive material quantities of concern. Order imposing increased controls (effective immediately). [Cited 23 Sept 2023]. Available at: http://pbadupws.nrc.gov/docs/ML0531/ML053130218.pdf.

[131] Department of Energy/National Nuclear Security Administration’s (DOE/NNSA) Office of Radiological Security (ORS). [Internet] 2021 cesium irradiator replacement project. [Cited 23 Sept 2023]. Available at: https://www.energy.gov/sites/default/files/2021-04/20210416%20-%20Cesium%20Irradiator%20Replacement%20Project.pdf.

[132] Demaria M, Ohtani N, Youssef SA, Rodier F, Toussaint W, Mitchell JR, Laberge RM, Vijg J, Van Steeg H, Dolle ME, Hoeijmakers JH, de Bruin A, Hara E, Campisi J (2014). An essential role for senescent cells in optimal wound healing through secretion of PDGF-AA. Dev Cell.

[133] Yabluchanskiy A, Tarantini S, Balasubramanian P, Kiss T, Csipo T, Fulop GA, Lipecz A, Ahire C, DelFavero J, Nyul-Toth A, Sonntag WE, Schwartzman ML, Campisi J, Csiszar A, Ungvari Z (2020). Pharmacological or genetic depletion of senescent astrocytes prevents whole brain irradiation-induced impairment of neurovascular coupling responses protecting cognitive function in mice. Geroscience.

[134] Li B, Yabluchanskiy A, Tarantini S, Allu SR, Sencan-Egilmez I, Leng J, Alfadhel MAH, Porter JE, Fu B, Ran C, Erdener SE, Boas DA, Vinogradov SA, Sonntag WE, Csiszar A, Ungvari Z, Sakadzic S (2023). Measurements of cerebral microvascular blood flow, oxygenation, and morphology in a mouse model of whole-brain irradiation-induced cognitive impairment by two-photon microscopy and optical coherence tomography: evidence for microvascular injury in the cerebral white matter. Geroscience..

[135] Chang J, Wang Y, Shao L, Laberge RM, Demaria M, Campisi J, Janakiraman K, Sharpless NE, Ding S, Feng W, Luo Y, Wang X, Aykin-Burns N, Krager K, Ponnappan U, Hauer-Jensen M, Meng A, Zhou D (2016). Clearance of senescent cells by ABT263 rejuvenates aged hematopoietic stem cells in mice. Nat Med.

[136] Zhu Y, Tchkonia T, Fuhrmann-Stroissnigg H, Dai HM, Ling YY, Stout MB, Pirtskhalava T, Giorgadze N, Johnson KO, Giles CB, Wren JD, Niedernhofer LJ, Robbins PD, Kirkland JL (2016). Identification of a novel senolytic agent, navitoclax, targeting the Bcl-2 family of anti-apoptotic factors. Aging Cell.

[137] Demaria M, O'Leary MN, Chang J, Shao L, Liu S, Alimirah F, Koenig K, Le C, Mitin N, Deal AM, Alston S, Academia EC, Kilmarx S, Valdovinos A, Wang B, de Bruin A, Kennedy BK, Melov S, Zhou D, Sharpless NE, Muss H, Campisi J (2017). Cellular senescence promotes adverse effects of chemotherapy and cancer relapse. Cancer Discov.

[138] Ueno M, Aoto T, Mohri Y, Yokozeki H, Nishimura EK (2014). Coupling of the radiosensitivity of melanocyte stem cells to their dormancy during the hair cycle. Pigment Cell Melanoma Res.

[139] Kudlova N, Slavik H, Duskova P, Furst T, Srovnal J, Bartek J, Mistrik M, Hajduch M (2021). An efficient, non-invasive approach for in-vivo sampling of hair follicles: design and applications in monitoring DNA damage and aging. Aging (Albany NY).

[140] Kinoshita K, Ishimine H, Shiraishi K, Kato H, Doi K, Kuno S, Kanayama K, Mineda K, Mashiko T, Feng J, Nakagawa K, Kurisaki A, Itami S, Yoshimura K. Cell and tissue damage after skin exposure to ionizing radiation: short- and long-term effects after a single and fractional doses. Cells Tissues Organs. 2014;200:240–52. 10.1159/000435809 26359658

[141] Endou M, Aoki H, Kobayashi T, Kunisada T (2014). Prevention of hair graying by factors that promote the growth and differentiation of melanocytes. J Dermatol.

[142] Tanimura S, Tadokoro Y, Inomata K, Binh NT, Nishie W, Yamazaki S, Nakauchi H, Tanaka Y, McMillan JR, Sawamura D, Yancey K, Shimizu H, Nishimura EK (2011). Hair follicle stem cells provide a functional niche for melanocyte stem cells. Cell Stem Cell.

[143] Aoki H, Hara A, Motohashi T, Kunisada T (2013). Keratinocyte stem cells but not melanocyte stem cells are the primary target for radiation-induced hair graying. J Invest Dermatol.

[144] Kowald A, Passos JF, Kirkwood TBL (2020). On the evolution of cellular senescence. Aging Cell.

[145] Baker DJ, Petersen RC (2018). Cellular senescence in brain aging and neurodegenerative diseases: evidence and perspectives. J Clin Invest.

[146] Kirkland JL, Tchkonia T (2017). Cellular senescence: a translational perspective. EBioMedicine.

[147] Wiley CD, Campisi J (2016). From ancient pathways to aging cells-connecting metabolism and cellular senescence. Cell Metab.

[148] Childs BG, Durik M, Baker DJ, van Deursen JM (2015). Cellular senescence in aging and age-related disease: from mechanisms to therapy. Nat Med.

[149] Campisi J (2013). Aging, cellular senescence, and cancer. Annu Rev Physiol.

[150] Victorelli S, Lagnado A, Halim J, Moore W, Talbot D, Barrett K, Chapman J, Birch J, Ogrodnik M, Meves A, Pawlikowski JS, Jurk D, Adams PD, van Heemst D, Beekman M, Slagboom PE, Gunn DA, Passos JF (2019). Senescent human melanocytes drive skin ageing via paracrine telomere dysfunction. EMBO J.

[151] Meador JA, Morris RJ, Balajee AS (2022). Ionizing radiation-induced dna damage response in primary melanocytes and keratinocytes of human skin. Cytogenet Genome Res.

[152] Kang C, Xu Q, Martin TD, Li MZ, Demaria M, Aron L, Lu T, Yankner BA, Campisi J and Elledge SJ. The DNA damage response induces inflammation and senescence by inhibiting autophagy of GATA4. Science. 2015;349:aaa5612. 10.1126/science.aaa5612 PMC4942138 26404840

[153] d'Adda di Fagagna F. Living on a break: cellular senescence as a DNA-damage response. Nat Rev Cancer. 2008;8:512–22. 10.1038/nrc2440 18574463

[154] Ahire C, Nyul-Toth A, DelFavero J, Gulej R, Faakye JA, Tarantini S, Kiss T, Kuan-Celarier A, Balasubramanian P, Ungvari A, Tarantini A, Nagaraja R, Yan F, Tang Q, Mukli P, Csipo T, Yabluchanskiy A, Campisi J, Ungvari Z, Csiszar A. Accelerated cerebromicrovascular senescence contributes to cognitive decline in a mouse model of paclitaxel (Taxol)-induced chemobrain. Aging Cell. 2023;22(7):e13832. 10.1111/acel.13832 PMC10352561 37243381

[155] Tarantini S, Balasubramanian P, Delfavero J, Csipo T, Yabluchanskiy A, Kiss T, Nyul-Toth A, Mukli P, Toth P, Ahire C, Ungvari A, Benyo Z, Csiszar A, Ungvari Z (2021). Treatment with the BCL-2/BCL-xL inhibitor senolytic drug ABT263/Navitoclax improves functional hyperemia in aged mice. Geroscience.

[156] Xu M, Pirtskhalava T, Farr JN, Weigand BM, Palmer AK, Weivoda MM, Inman CL, Ogrodnik MB, Hachfeld CM, Fraser DG, Onken JL, Johnson KO, Verzosa GC, Langhi LGP, Weigl M, Giorgadze N, LeBrasseur NK, Miller JD, Jurk D, Singh RJ, Allison DB, Ejima K, Hubbard GB, Ikeno Y, Cubro H, Garovic VD, Hou X, Weroha SJ, Robbins PD, Niedernhofer LJ, Khosla S, Tchkonia T, Kirkland JL (2018). Senolytics improve physical function and increase lifespan in old age. Nat Med.

[157] Roos CM, Zhang B, Palmer AK, Ogrodnik MB, Pirtskhalava T, Thalji NM, Hagler M, Jurk D, Smith LA, Casaclang-Verzosa G, Zhu Y, Schafer MJ, Tchkonia T, Kirkland JL, Miller JD (2016). Chronic senolytic treatment alleviates established vasomotor dysfunction in aged or atherosclerotic mice. Aging Cell.

[158] Adav SS, Ng KW (2023). Recent omics advances in hair aging biology and hair biomarkers analysis. Ageing Res Rev.

[159] Qiu R, Qiu X, Su M, Sun M, Wang Y, Wu J, Wang H, Tang D, Tao S (2023). Dietary restriction delays but cannot heal irradiation-induced hair graying by preserving hair follicle stem cells in quiescence. Rejuvenation Res..

